# Ruptured Intracranial Mycotic Aneurysm in Infective Endocarditis: A Natural History

**DOI:** 10.1155/2010/168408

**Published:** 2010-09-22

**Authors:** Isabel Kuo, Theodore Long, Nathan Nguyen, Bharat Chaudry, Michael Karp, Nerses Sanossian

**Affiliations:** ^1^University of Southern California Keck School of Medicine, Los Angeles, CA 90089, USA; ^2^Division of Geriatric, Hospitalist, Palliative, and General Internal Medicine, Department of Medicine, LAC+USC Medical Center, University of Southern California Keck School of Medicine, 1200 N. State St. IRD 310, Los Angeles, CA 90033, USA; ^3^Department of Neurology, LAC+USC Medical Center, University of Southern California Keck School of Medicine, Los Angeles, CA 90033, USA

## Abstract

Mycotic aneurysms are a rare cause of intracranial aneurysms that develop in the presence of infections such as infective endocarditis. They account for a small percentage of all intracranial aneurysms and carry a high-mortality rate when ruptured. The authors report a case of a 54-year-old man who presented with infective endocarditis of the mitral valve and acute stroke. He subsequently developed subarachnoid hemorrhage during antibiotic treatment, and a large intracranial aneurysm was discovered on CT Angiography. His lesion quickly progressed into an intraparenchymal hemorrhage, requiring emergent craniotomy and aneurysm clipping. Current recommendations on the management of intracranial Mycotic Aneurysms are based on few retrospective case studies. The natural history of the patient's ruptured aneurysm is presented, as well as a literature review on the management and available treatment modalities.

## 1. Introduction

Mycotic aneurysms (MAs), also known as infective or microbial aneurysms, are rare inflammatory neurovascular lesions that account for 0.7–6.5% of all intracranial aneurysms [1]. MAs are unique in their natural history and pathologic findings, with distinct angiographic features, and frequently develop at terminal arterial branches. Because their spontaneous rupture results in subarachnoid and intracerebral hemorrhage, they are associated with significant morbidity and mortality, as high as 60%–90% in earlier case studies, and 12–32% in more recent literature reviews [1, 2]. The following is a case report coupled with a methodical review of the presentation, diagnosis, complications, and management of MAs.

## 2. Case History

A 54-year-old Caucasian male with a history of IE complicated by cerebrovascular accident 15 years prior presented to the emergency department complaining of slurred speech for the past two days. He reported that his lower mouth felt as though it had been “numbed by a dentist,” resulting in subjective dysarthria. At the time of presentation, he denied headaches, visual changes, nausea, vomiting, or numbness or weakness in the extremities. 

 The patient was febrile at 102.3 degrees Fahrenheit, and his cardiovascular exam revealed a 3/6 holosystolic murmur most prominent at the left midclavicular line with radiation to the axilla. The neurologic exam was significant for left facial weakness, subtle slurring of speech, and diffuse hyperreflexia of all four extremities. Marked clubbing of his fingers on both hands was appreciated. 

**Figure 1:**
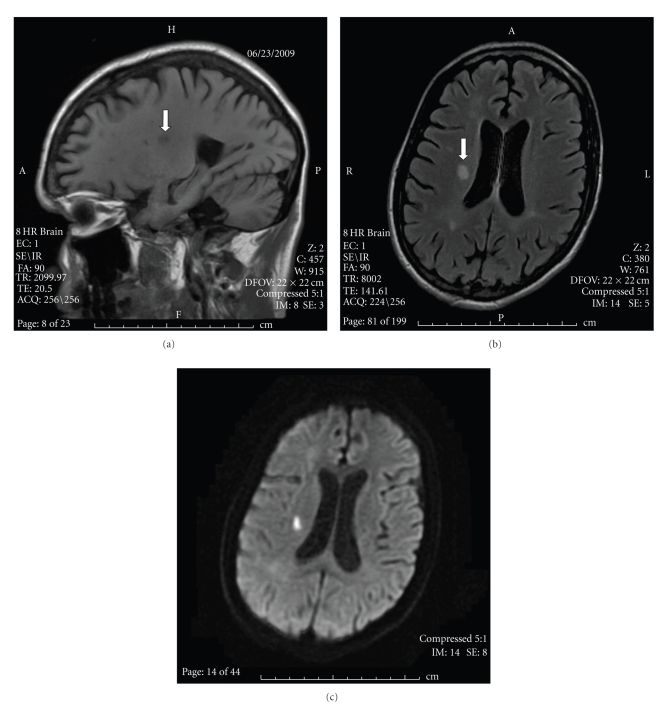
MRI of brain on presentation: (a) sagital view showing acute right side frontal lobe infarct marked by arrow. (b) Axial view with same right side frontal lobe infarct marked by arrow. (c) Diffusion-Weighted Image of ischemic stroke in the right centrum semiovale.

**Figure 2:**
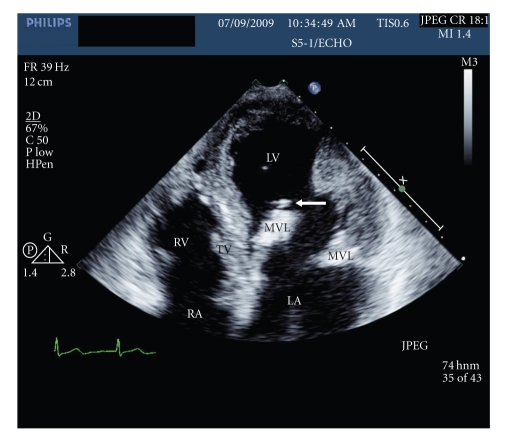
Four chamber view echocardiogram showing large vegetation on the MVL marked by arrow. LV-left ventricle, LA-left atrium, RV-right ventricle, RA-right atrium, TV-tricuspid valve, MVL-mitral valve leaflet.

 The head CT showed an old left posterior cerebral artery infarct and prominent basal ganglia calcification, but neither active bleeding nor acute infarction. The MRI was significant for a small acute nonhemorrhagic infarct in the right frontal periventricular white matter as well as scattered punctate areas of chronic blood products (Figure 1). The echocardiogram visualized large mobile vegetations on the mitral valve (Figure 2). 6 of 6 blood cultures were positive for *Streptococcus viridans*. Intravenous Penicillin G and Gentamicin were started immediately, and after 5 days of antibiotic treatment, the patient's dysarthria and left facial weakness improved. 

**Figure 3:**
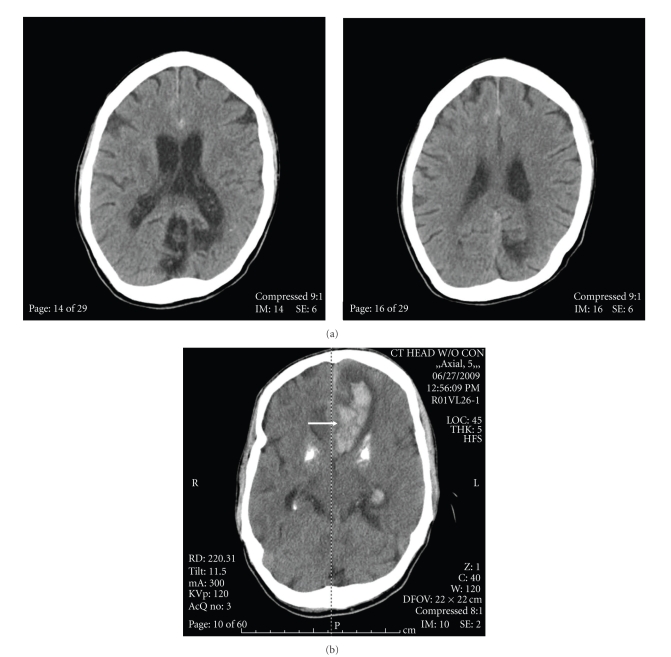
Progression of the ruptured Mycotic Aneurysm: (a) initial subarachnoid hemorrhage after 6 days IV antibiotics. Note interhemispheric hemorrhage. (b) Repeat noncontrast CT Brain 12 hours later, intraparenchymal hemorrhage is marked by arrow. Note prominent basal ganglia calcifications.

 Six days into antibiotic therapy, the patient complained of a severe headache and was noticeably slow to respond. The slurred speech and left facial weakness also returned. A noncontrast CT head was repeated and showed acute subarachnoid hemorrhage (Figure 3). The patient's condition deteriorated; he became obtunded and required endotracheal intubation and mechanical ventilation. 

**Figure 4:**
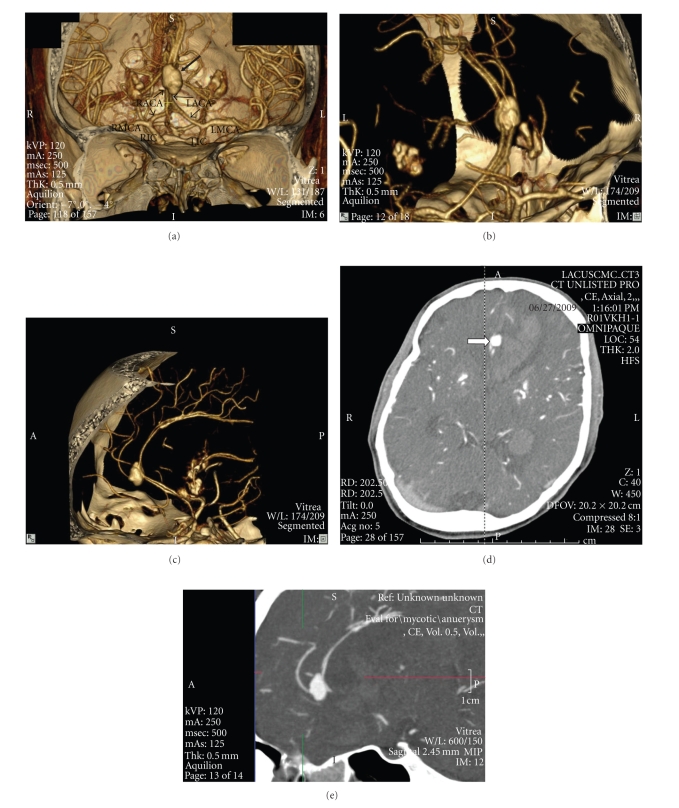
CT Cerebral angiogram showing a large fusiform aneurysm at the distal anterior cerebral artery (ACA). (a) Shaded Surface Display CT coronal view showing a large fusiform aneurysm at the distal segment of the ACA marked by the large arrow. Proximal ACA segments are marked by the smaller arrows. RIC: right internal carotid artery, LIC: left internal carotid artery, RMCA: right middle cerebral artery, LMCA-left middle cerebral artery, RACA and LACA are right and left anterior cerebral arteries, respectively. (b) Close-up coronal view of ACA mycotic aneurysm. (c) Additional sagital view of mycotic aneurysm. (d) CT Angiogram axial view, an arrow marks the location of the ruptured aneurysm adjacent to a large hemorrhagic lesion. (e) CT Angiogram sagital view demonstrating areas of intracerebral hemorrhage.

 The subsequent CT Cerebral Angiogram established a multilobulated cerebral aneurysm at the A3 segment of the right distal anterior cerebral artery measuring 13 mm by 8.7 mm at the coronal plane, as well as interval development of intraventricular and intraparenchymal hemorrhage (Figure 4). After the placement of a ventriculostomy catheter, the patient was emergently taken to the operating room to undergo aneurysm clipping and ligation. The aneurysm was located during dissection of the falx interhemispheric fissure. As is typical of a mycotic aneurysm, it was of poor consistency with degenerated and friable walls. The operation was complicated by aneurysm rupture for which hemostasis was achieved with microcottonoids and clipping. During the recovery period the patient was eventually able to follow basic commands, though the extent of his intracranial and valvular disease was severe. He ultimately required a tracheostomy, and percutaneous gastric tube. Because of the presence of intracranial hemorrhage and acute infarct, replacement of his mitral and aortic valves was deferred. The patient was transferred to a long-term nursing facility and given followup with the cardiothoracic surgery team for reassessment after a 6 week course of IV antibiotics. 

## 3. Discussion

### 3.1. Pathogenesis

Mycotic aneurysms develop in IE when friable cardiac vegetations give rise to septic emboli that lodge in intracranial vessels at branching points and distal branches. These emboli may occlude vessels, cause cerebral infarction, or promote infection [3]. 

 The vasa vasorum theory is the most widely accepted mechanism of pathogenesis. It proposes that bacteria from septic emboli escape through the vasa vasorum and cause severe inflammation of the adventitia. The infection then spreads inwardly [4–6]. The arterial pulsation against the weakened vessel wall eventually results in aneurysm formation and enlargement. The resulting aneurysms are usually fusiform and eccentric, without saccular characteristics, and are more common in the anterior circulation [7]. 

Histologically, MAs are characterized by acute neutrophilic infiltration, along with marked intimal proliferation and internal elastic lamina destruction. The responsible organism may be identified with appropriate staining [1]. Although a wide variety of bacteria, mycobacteria, viruses, and fungi may cause mycotic aneurysms, *Viridans Streptococci *and *Staphylococcus aureus *are the most common etiologic organisms [1].

### 3.2. Clinical Presentation and Diagnosis

In a large case series by Kannoth et al. 25 patients with infectious intracranial aneurysms presented with initial symptoms of headache (83%), fever (67%), vomiting (50%), ocular palsy (25%), seizures (21%), behavioral changes (21%), hemiparesis (21%), drowsiness (17%), and loss of consciousness (17%) [8]. Almost half the patients met Duke's criteria for IE and gave a history of either rheumatic or congenital heart disease. 

 A scoring system based on the presence of specific clinical and radiographic findings has been proposed for the diagnosis of MA. Points are given for the presence of clinical markers, such as IE, meningitis, orbital cellulitis, cavernous sinus thrombophlebitis, persistent fever, age less than 45, recent lumbar puncture, and radiographic evidence of aneurysm multiplicity, distal location, fusiform shape, and change in size [9]. Applying this scheme to our patient prior to CT Angiography would yield a score of 2. The sensitivity and specificity of this value are 100% and 87.4%, respectively.

### 3.3. Radiographic Imaging Modalities

Cerebral vascular imaging is available through CT Angiogram (CTA), Magnetic Resonance Angiogram (MRA), and Digital Subtraction Angiography (DSA). DSA, until recent decades, was the gold standard in intracranial aneurysm diagnosis [10]. The advent of Multidetector CT imaging (MDCT) has increased the resolution of CTA, allowing for complete visualization of the intracranial vascular tree. This imaging modality carries a lower contrast burden and risk of permanent neurologic deficits than DSA. When compared to DSA, CTA had a sensitivity of 90% and specificity of 86% in recent systematic meta-analysis [11]. MRA is also an emerging imaging modality in the diagnosis of intracranial aneurysms. When compared to CTA, it is 87% sensitive and 95% specific; however for aneurysms smaller than 3 mm, its sensitivity falls to 38% versus 61% for CTA.

### 3.4. Management and Treatment

Given the relative rarity of this disease, current recommendations regarding the management and treatment of MA have largely been restricted to a limited number of case reports. Over the past decade, the management of infective intracranial aneurysms has been divided into medical, endovascular, and surgical treatment. The medical intervention that is uniformly recommended is long- term intravenous antibiotic therapy for at least 6 weeks. In 1984, Morawetz and Karp observed that unruptured MAs could undergo spontaneous thrombosis, suggesting that MAs could resolve completely with antibiotic therapy alone [3]. In a review of 20 cases of MAs over a ten-year period by Chun in 2001, seven patients were initially treated conservatively with IV antibiotics alone and followed by serial angiography [12]. In this series, the aneurysms in two patients decreased in size, one did not change, two achieved successful thrombosis, and the remaining two enlarged. Based on this review, the conclusion regarding unruptured MAs is that medical management with 6 weeks of IV antibiotic therapy is reasonable if closely followed by serial angiography. The goal of serial angiography would be to demonstrate improvement in aneurysm size and resolution. Zhao et al. reported a case in which a MA failed to show radiographic decrease in size after 2 weeks of medical therapy, and was successfully treated with endovascular therapy [13]. 

Endovascular therapy has rapidly evolved in its efficacy and ability to access more distal aneurysms. The safety profile of this intervention is difficult to interpret, as it is based only on anecdotal and case-report data. A meta-analysis was performed on 16 patients in previously published cases who underwent endovascular treatment; 69% had a good outcome, while none had procedural-related complications [12]. However, parent artery sacrifice was much more common in patients who underwent endovascular treatment versus open craniotomy with surgical ligation. This affects the management of diseased vessels supplying eloquent brain parenchyma, such as areas that involve language, sensorimotor cortex, visual cortex, hypothalamus, thalamus, cerebral peduncles, and brain stem. 

 In a retrospective review performed on 14 patients with intracranial MAs who underwent endovascular intervention at between 1991 and 1999 at a major French hospital [14], no deaths or complications were reported, 11 of the 14 patients showed stable lesions on followup angiography 6–24 months after endovascular embolization, and 9 had complete resolution of their presenting neurologic deficit [14]. Endovascular therapy is potentially safer and more effective than open craniotomy if performed at a high volume tertiary center and in the absence of increased ICP, mass effect, hypotension, hematoma, or involvement of eloquent territory. 

 Surgical therapy with open craniotomy and aneurysm clipping is reserved for patients with intraparenchymal hemorrhage, or for those who require clot evacuation and emergent reversal of increasing intracranial pressure. Another distinct advantage of surgical intervention is the option of vascular bypass to preserve distal blood flow, which is a vital consideration when the aneurysm involves eloquent territory. 

### 3.5. Valvular Repair in the Setting of Intracranial Septic Emboli

The purpose of cardiac surgery is to remove the source of cerebral emboli and to improve hemodynamics [3]. Whether the aneurysm has ruptured influences the timing and sequence of cardiothoracic and neurosurgery [1]. 

 Cardiac surgery is relatively safe if the MA is unruptured. Cardiopulmonary bypass, even with its requirement for heparinization, does not impose additional risks of rupture perioperatively [15]. In the absence of a hemorrhagic infarct, valve replacement can be performed with very little risk of perioperative stroke [16]. However, if the MA has ruptured and there is mass effect from an intracerebral hematoma or abscess, the intracranial aneurysm should be repaired first [3]. In the presence of cerebral infarction, the danger of hemorrhagic transformation warrants a 2-3 week postponement of cardiac surgery [1]. The exception to this rule would be if left heart failure were to develop as a consequence to IE. The presence of left heart failure would compromise cerebral perfusion, thus negating the benefits of any prior interventions performed on an intracranial MA. Hashimoto et al. reported a case in which a 24-year-old female with mitral valve IE and ruptured intracranial MA, safely underwent urgent mechanical mitral valve replacement for worsening congestive heart failure 10 days after suffering from an intracerebral hemorrhage. Neither the IE nor MA recurred in the postoperative 4 year followup period [17]. Shiraishi et al. reported a similar case in a 55 year old patient who presented with ruptured MA and left heart failure from aortic valve IE. In this case the patient underwent emergent bioprosthetic aortic valve replacement and did well with resolution of MA 9 months postoperatively [18].

### 3.6. Prognosis and Outcome

The general consensus in the current literature is that ruptured MAs carry a worse prognosis than unruptured MAs; however, the rarity of intracranial MAs makes it difficult to ascertain prognostic markers that may help predict likely outcomes. A comprehensive review of 27 clinical series and 287 patients with intracranial MA diagnosed between 1950–2009 was recently conducted by Ducruet et al. The analysis, while limited due to heterogeneity of presentations and variable followup duration, concluded that in all treatment modalities combined, 62% of patients had a positive outcome, 20% faced further neurological decline, 5% died before an invasive intervention could be performed, and 12% died immediately after an intervention was performed whether surgical or endovascular [19].

## 4. Summary

In the case of our patient, the diagnosis of an acute stroke was made early in the hospital course but a MA was not considered in the differential until after the patient had a subarachnoid hemorrhage. MA can be prevented from rupture when they are suspected and treated early. This case emphasizes the importance of considering the presence of MA in the differential diagnosis in the setting of suspected endocarditis and focal neurological deficits. If this constellation is present, MA should be investigated with CT Angiography. Frequent evaluations of neurological status should be performed, and a low threshold of suspicion for subarachnoid hemorrhage should be maintained. Finally, a neurosurgical consult should be obtained to determine the appropriate therapeutic intervention once the diagnosis has been made. 

 Medical management should be reserved for patients with aneurysms that have not yet ruptured and should be followed closely with serial cerebral angiograms. Endovascular intervention should be based on the stability of the patient and involvement of eloquent territory. Surgical treatment should be performed emergently in the setting of intraparenchymal hemorrhage or increased intracranial pressure.

## Figures and Tables

**Figure 5:**
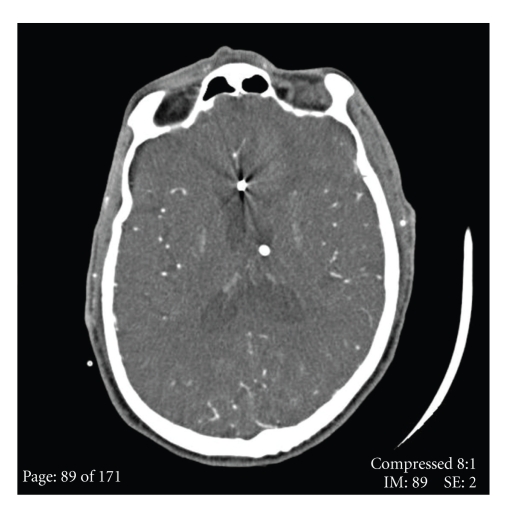
One week postoperative CT imaging demonstrating a metallic clip in the ACA, and a ventriculostomy in the lateral ventricle.
